# Features and Functionalities of Smartphone Apps Related to COVID-19: Systematic Search in App Stores and Content Analysis

**DOI:** 10.2196/20334

**Published:** 2020-08-25

**Authors:** Roberto Collado-Borrell, Vicente Escudero-Vilaplana, Cristina Villanueva-Bueno, Ana Herranz-Alonso, Maria Sanjurjo-Saez

**Affiliations:** 1 Hospital General Universitario Gregorio Marañón Madrid Spain

**Keywords:** COVID-19, mobile apps, contact tracing, monitoring, telemedicine, smartphone

## Abstract

**Background:**

Knowledge of the quantity and quality of apps related to coronavirus disease (COVID-19) is lacking. In addition, no directory has been established listing all the apps developed to address the COVID-19 pandemic.

**Objective:**

The aim of this study was to identify smartphone apps designed to address the COVID-19 pandemic and to analyze their characteristics.

**Methods:**

We performed an observational, cross-sectional, descriptive study of all smartphone apps associated with COVID-19. Between April 27 and May 2, 2020, we searched the App Store (iOS) and Google Play Store (Android) for COVID-19 apps. The search terms used were *coronavirus*, *COVID-19*, and *SARS-COV-2*. The apps were downloaded and evaluated. The variables analyzed were name, platform, country, language, category, cost, update date, size, version, number of downloads, developer, and purpose. Purpose was further classified into the following categories: news, general information, self-diagnosis, contact tracing, notices to contacts, notification of close cases, awareness, helplines, monitoring of clinical parameters, recording of symptoms and treatment, and messaging with health care professionals.

**Results:**

We identified 114 apps on the investigated platforms. Of these, 62/114 (54.4%) were on Android and 52/114 (45.6%) were on iOS. Of the 114 apps, 37 (32.5%) were developed in Europe, 32 (28.1%) in Asia, and 30 (26.3%) in North America. The most frequent languages were English (65/114, 57.0%), Spanish (34/114, 29.8%), and Chinese (14/114, 12.3%). The most common categories were health and well-being/fitness apps (41/114, 41.2%) and medicine apps (43/114, 37.7%). Of the 114 apps, 113 (99.1%) were free. The mean time between the date of the analysis and the date of the last update was 11.1 days (SD 11.0). Overall, 95 of the 114 apps (83.3%) were intended for the general population, 99 apps (7.9%) were intended for health professionals, and 3 apps (2.6%) were intended for both. Regarding the type of developer, 64/114 apps (56.1%) were developed by governments; 42/114 (64.1%) were developed by national governments, and 23/114 (35.9%) were developed by regional governments. The apps with the highest number of downloads (100,000+) were developed by governments (*P*=.13), except for the World Health Organization app (500,000+). The purposes of the apps available in Western languages (107/114, 93.9%) were determined; the most common purposes were general information about COVID-19 (66, 64.0%), COVID-19 news (53, 51.0%), recording of symptoms (53, 51.0%), and contact tracing (51, 47.7%). More than one purpose was identified for 99/107 apps (92.5%).

**Conclusions:**

This paper offers a comprehensive and unique review of all available COVID-19 apps. Governments have adopted these tools during the pandemic, and more than half of the apps were developed by government agencies. The most common purposes of the apps are providing information on the numbers of infected, recovered, and deceased patients, recording of symptoms, and contact tracing.

## Introduction

The novel coronavirus SARS-CoV-2, which causes coronavirus disease (COVID-19), has created a health care crisis that is unprecedented in our recent history. Since the first case was detected in late 2019 [1], more than 4 million infections and more than 300,000 deaths have been confirmed worldwide [2]. These data, together with the high capacity for progression of COVID-19 and the lack of knowledge about its behavior patterns, highlight the urgent need to find a solution to this health emergency [2].

Governments, companies, and several public and private institutions worldwide are combining their efforts to find an effective solution to reduce the risk of spreading COVID-19 [3]. In this scenario, digital technologies constitute essential tools for improving the health of populations as well as basic services provided to them [4,5]. The World Health Organization (WHO) recently published 10 recommendations for improving population health and essential services through digital technologies [6].

It is currently estimated that more than 5 billion people use mobile phones [7]; moreover, according to the “State of Mobile in 2019” report published in 2018 [8], 194 billion apps have been downloaded worldwide. Apps are therefore easily accessed and implemented by the vast majority of the world’s population [9]. Apps have various beneficial functions related to the COVID-19 pandemic, from remote follow-up by health care professionals to monitoring the number of infections. One method of monitoring the number of infections is contact tracing [10-13]. According to Yasaka et al [14], smartphone-based contact tracing presents a novel solution that preserves privacy and has the potential to suppress an epidemic or pandemic. Another scenario in which apps may be useful is self-diagnosis, in which an app can process a patient’s information and provide an approximate diagnosis [4]. Taking these data into account, apps can be useful and effective tools for managing the COVID-19 pandemic.

The success of an app depends on the context in which it is used and the appropriateness of its design. However, the current lack of knowledge about the quantity and quality of apps related to COVID-19 is compounded by the lack of directories listing the apps that have been developed to address the pandemic.

The objective of this study was to identify smartphone apps designed to address the COVID-19 pandemic and to analyze their characteristics.

## Methods

We performed an observational, cross-sectional, descriptive study of all smartphone apps associated with COVID-19 available on the iOS and Android platforms.

The methodology used for the selection of the apps was based on the Preferred Reporting Items for Systematic Reviews and Meta-Analyses (PRISMA) system. Between April 27 and May 2, 2020, we searched the App Store (iOS) and the Google Play Store (Android) for all COVID-19–related apps. The search terms were *coronavirus*, *COVID-19*, and *SARS-COV-2*, and no language restrictions were applied. After identification, the information available on each platform was analyzed, and all apps were downloaded and evaluated. The apps included in the App Store were downloaded onto an iPhone 8 (iOS version 12.3.2), and the apps found in the Google Play Store were downloaded onto a Xiaomi Mi A1 (Android version 9.0).

The variables analyzed were the app name, platform (Android or iOS), country of origin, language, category, cost, date of last update, size, version, number of downloads, developer, and purpose. Purpose was further classified into the following categories: news, general information, self-diagnosis, contact tracing, alerts to contacts, notification of closed cases, awareness, helplines, monitoring of clinical parameters (temperature, blood pressure, heart rate, respiratory frequency, oxygen saturation), symptom log, treatment log, and messaging with health care professionals. “Contact tracing” was defined as the ability of the app to identify the locations of COVID-19 cases and thus to control the spread of the virus.

The app was evaluated by two independent researchers with experience in app analysis, design, and development (RC and VE). Continuous variables were compared using the *t* test when the distribution was normal or the Mann-Whitney test when it was not. Categorical variables were compared using an uncorrected chi-square test or Fisher exact test. The Cohen κ test was performed to guarantee the reliability of the data analyzed by the two independent researchers. Data were analyzed using Stata version IC-14 (descriptive statistics). A *P* value <.05 was considered statistically significant.

## Results

### App Characteristics

We identified 114 apps: 62/114 (54.4%) were on Android and 52/114 (45.6%) were on iOS. 50/114 apps (43.9%) were available on both platforms. Supplementary Table 1 (Multimedia Appendix 1) shows the names of the apps classified according to the country in which they were developed. Of the 114 apps, 37 (32.5%) were developed in Europe, 32 (28.1%) in Asia, 30 (26.3%) in North America, 12 (10.5%) in Central America–South America, and 3 (2.6%) in Africa. The most common languages were English (65/114 apps, 57.0%), Spanish (34 apps, 29.8%), and Chinese (14 apps, 12.3%). The average number of languages per app was 1.9 (range 1-12). Seven of the 114 analyzed apps (6.1%) were only available in Asian languages.

The most frequent categories of the 114 apps were health and well-being/fitness (47 apps, 41.2%), medicine (43 apps, 37.7%), utilities (5 apps, 4.4%), education (3 apps, 2.6%), and lifestyle (3 apps, 2.6%). A full list of the categories is shown in Table 1. Regarding cost, 113/144 apps (99.1%) were free. At least one version update was found for 96/114 apps (86.8%). Among the 96 apps that updated, 90 (93.8%) updated in April and 6 (6.3%) updated in March. The average time between the date of analysis and the date of the last update was 11.1 days (SD 11.0). The average size of the apps was 27.3 MB (SD 28.9). Regarding content classification, 85 of the 114 apps (74.6%) were intended for all age groups, 12 (10.5%) for people older than 17 years, and 9 (7.9%) for people older than 12 years; 8 (7.0%) did not specify an age group. The target populations of the 114 apps were the general population (95 apps, 88.8%), health professionals (9 apps, 78.4%), and both (3 apps, 2.8%).

Regarding the type of developer, 64/114 apps (56.1%) were developed by governments. Of these, 42/114 (64.1%) were national governments, and 22 (35.9%) were regional. A full list of developers is shown in Table 2.

**Table 1:** Categories of the apps (N=114).

Category	n (%)
Health and well-being/fitness	47 (41.2)
Medicine	43 (37.7)
Utilities	5 (4.4)
Education	3 (2.6)
Lifestyle	3 (2.6)
Communication	2 (1.8)
Reference	2 (1.8)
News	2 (1.8)
Productivity	2 (1.8)
Parental control	1 (0.9)
Electronic health administration and medicine	1 (0.9)
Business	1 (0.9)
Economy and business	1 (0.9)
Travel and guides	1 (0.9)

**Table 2:** Developers of the apps (N=114).

Developer	n (%)
Government	64 (56.1)
Health-related technology company	19 (16.7)
Nonhealth-related technology company	11 (9.6)
Hospital	7 (6.1)
University	4 (3.5)
Independent health professionals	3 (2.6)
Not specified	2 (1.8)
World Health Organization	1 (0.9)
University or hospital	1 (0.9)
Voluntary humanitarian institution or public interest	1 (0.9)
Foundation	1 (0.9)

The numbers of downloads of the apps were determined where available (this information is provided on the Google Play store, but not the App Store). The median number of downloads of the apps was 5000 (range 1 to 50,000,000+). The apps with the most downloads (100,000+) were developed by governments (*P*=.13), except for the WHO app (500,000+). The app with the highest number of downloads (50,000,000+) was developed by the Indian government, followed by apps developed by the Polish and Colombian governments (1,000,000+). Supplementary Table 2 (Multimedia Appendix 1) shows the apps developed by various governments and the number of times they were downloaded.

Regarding purpose, we did not find a statistically significant relationship between the number of purposes and the number of downloads (*P*=.06). Analysis of the purpose of the 107 apps available in Western languages revealed that 99 apps (92.5%) had more than 1 purpose. The most frequent purposes were general information about the coronavirus pandemic (66/107 apps, 64%), news (53/107 apps, 51.0%), recording of symptoms (53/107 apps, 51.0%), and contact tracing (51/107 apps, 47.7%). The remaining purposes are shown in Table 3.

**Table 3:** Purposes of the apps (n=107).

Purpose	n (%)
General information about COVID-19^a^	66 (64.0)
News	53 (51.0)
Recording of symptoms	53 (51.0)
Contact tracing	51 (47.7)
Awareness	46 (43.0)
Helplines	46 (43.0)
Self-diagnosis	37 (34.6)
Warning of nearby cases	28 (26.2)
Contact with health professionals	22 (20.6)
Monitoring of clinical parameters	13 (12.1)
Recording treatments	6 (5.6)
Access control	4 (3.7)
Mobile contact warning	5 (4.7)

^a^COVID-19: coronavirus disease.

Apps developed for the purposes of contact tracing (19 apps, 18.4%), notifications of close cases (10 apps, 9.7%), access control (3 apps, 2.9%), and notice to contacts of the mobile (2 apps, 1.9%) were more common in Asia (*P*=.06). The purposes of general information (23 apps, 22.3%), news (17 apps, 16.5%), recording of symptoms (16 apps, 15.5%), and contact with health care professionals (8 apps, 7.8%) were more common among the apps developed in Europe. The most frequently observed functionalities of the apps developed in North America were general information (15 apps, 14.6%), and recording of symptoms (14 apps, 13.6%). In Central America–South America, the purposes of the apps were general information (10 apps, 9.3%) and news (10 apps, 9.3%) (*P*=.06). The Cohen κ coefficient was .96, indicating a degree of agreement of 96%.

## Discussion

### Principal Findings and Comparison With Prior Work

We provide a comprehensive and unique review of all available COVID-19–related apps. This is especially important because due to recently heightened security measures of iOS and Google with respect to COVID-19–related apps that do not come from official sources, there is currently no site where all available apps can be consulted [15]. In the Google Play Store, it is very difficult to find apps related to COVID-19 from countries other than that in which the user resides.

The present study, in which a total of 114 apps are analyzed, underlines the high number of apps designed by governments; this contrasts with other app reviews related to health, in which the leading developer is usually a scientific foundation or hospital [16,17]. Mobile health (mHealth) has evolved in recent years to position itself as a critical tool in patient communication and monitoring. However, the current pandemic has proven to be a turning point for mHealth technologies. Due to the rapid expansion of COVID-19 and its transmission by asymptomatic patients, detecting and isolating positive cases of COVID-19 has become a priority [3]. The ease of use of mobile phones and their accessibility by the majority of the world’s population, added to the need for information from users, avoidance of travel and contact, and identification of cases, has positioned apps as critical tools in the management of the coronavirus pandemic. In this sense, the governments of the most affected countries have opted to implement app-based initiatives that collect user data, helping to increase knowledge about the prevention, detection, and monitoring of COVID-19 [3,5,18]. These apps have been designed in record time, taking into account that the average time to develop an app is estimated to be one year [19].

The purposes of the leading apps developed by governments include general information, news about the pandemic, and monitoring of the areas affected by the virus. The latter type of app can be used to collect specific sociodemographic information and trace contacts. Many apps are based on a GPS system through which users can determine whether they have traveled through places where cases of COVID-19 were previously detected, as well as the date on which the contagion was confirmed [13,14,18]. Contact tracing has been used successfully for other infections, such as tuberculosis and Ebola virus [4]. According to Yasaka *et al* [14], contact tracing is a viable solution to limit the transmission of disease. Because half of infections occur before the patient develops symptoms, a geolocation system can help users avoid critical areas and possible infection. These apps developed by governments were the most frequently downloaded. We did not find any relationship between the number of apps developed in a country or continent and the severity of the disease in that location. For example, apps have been developed by countries with early COVID-19 outbreaks (eg, Italy or Spain), later outbreaks (eg, Colombia or Argentina), or even countries where infection rates are relatively low (eg, Poland).

Data protection issues are a key problem with this type of app. While apps developed in Western countries collect most data anonymously and in aggregate, some apps in Far Eastern countries collect greater amounts of personal data, enabling authorities to take more active measures to control the spread of the disease [11,12,18,20]. iOS and Google are currently working on a joint project to use people’s mobility data to better monitor and predict COVID-19 infections while ensuring that users’ privacy is respected [20].

Unlike Asia, in Europe and North America, the most common purposes of the apps are providing information on the numbers of persons infected, recovered, and deceased, enabling the recording of symptoms, and providing contact with health professionals. The number of apps related to symptom recording and contact with health care providers contrasts with data previously published by our group [16], which showed that only 6% of apps had this function. Recording of symptoms and communication between health care professionals and patients have been shown to improve access to health systems, reduce the use of health care resources, and improve monitoring and control of symptoms [16,19,21]. We also observed a high number of apps for self-diagnosis; the primary objective of these apps is to decongest health centers, thus enabling rapid and safe diagnosis of a larger number of people [14,22]. Digital/virtual triage apps enable the user to be evaluated for possible COVID-19 symptoms [4]. These systems are based on a series of questions related to the most frequent symptoms, such as cough, fever, dyspnea, and muscle pain. They also take into account whether an individual has been in contact with other people affected by COVID-19. Once the information has been processed, the app issues an approximate diagnosis. In the case of a positive result, the app itself shows a series of recommendations.

Medical follow-up apps have been designed in parallel for people who are self-isolating at home. According to Alwashmi et al [4], these apps have the potential to reduce transmission by minimizing physical contact between physician and patient. Through the recording and monitoring of the clinical parameters of COVID-19 (eg, temperature, oxygen saturation, and heart rate), together with virtual contact with health care professionals, the apps will enable people to adapt to the needs created by this situation, thus ensuring that patients receive medical attention.

Our Cohen κ analysis revealed an agreement of 96% between the two independent researchers, highlighting the robustness of our results.

### Limitations

Our study is limited by its cross-sectional nature, which means that any information reported here could become outdated as the COVID-19 situation evolves. However, because there is currently no repository of all available COVID-19–related apps, this review ranks as the most current source.

### Conclusions

Ease of access by the population and the use of artificial intelligence position apps as tools capable of detecting new foci of COVID-19 transmission, analyzing the rate of spread, monitoring possible symptoms, and approximately diagnosing positive cases at a distance. During the current pandemic, many governments have decided to implement apps to help curb the rapid expansion of COVID-19.

## Appendix

Multimedia Appendix 1 Supplementary tables.

## Abbreviations

COVID-19: coronavirus disease
mHealth: mobile health
PRISMA: Preferred Reporting Items for Systematic Reviews and Meta-Analyses
SARS-CoV-2: severe acute respiratory syndrome coronavirus 2
WHO: World Health Organization
