# Supplementary material for: Features and Functionalities of Smartphone Apps Related to COVID-19: Systematic Search in App Stores and Content Analysis
Source: J Med Internet Res. 2020 Aug 25;22(8):e20334. doi: 10.2196/20334 (PMC7479586; doi:10.2196/20334)
Supplement: Multimedia Appendix 1 [file jmir_v22i8e20334_app1.docx]

**Supplementary Table 1.** Apps related to coronavirus disease (COVID-19) identified in the study, sorted by country of origin.

| **NORTH AMERICA** | **EUROPE** | **ASIA** |
| --- | --- | --- |
| **Canada** | **Germany** | **Saudi Arabia** |
| Managing your stress & anxiety | OpenWHO: Knowledge for Health Emergencies | أمان - الأردن كوفيد-١٩ |
| BC COVID-19 Support | H.A.R.D. - COVID-19 | **Armenia** |
| Spectrum - Clinical Decisions | COVID-19 Symptom Tracker | Covid-19 Armenia |
| Canada COVID-19 | CoVive: your COVID-19 app | **Bahrein** |
| COVID-19 Resource for Midwives | **Austria** | BeAware Bahrain |
| **United States of America** | CoronaReport - COVID-19 reports for Social Science | **Korea** |
| patientMpower for COVID-19 USA | Stopp Corona | 자가격리자 안전보호 |
| Kencor COVID-19 | MyAus COVID-19 | **United Arab Emirates** |
| Sentinel Monitor (COVID-19 Management) | **Spain** | COVID19 UAE |
| BeWellXcel | GVA Responde | TraceCovid |
| NIOSH PPE Tracker | COVID-19.eus | Public Access Control System |
| CDC | Tonic: Medicina para todos | **Georgia** |
| PreMedicus | AyudaCOVID | Stop Covid |
| corona - care | GVA Coronavirus | **India** |
| Apollo COVID-19 | CONFINAPP | mCOVID-19 |
| CoronaFACTS | STOP COVID19 CAT | COVID-19 Tracker by Medinin |
| MUSC COVID-19 Vital Link | CoronaMadrid | Aarogya Setu |
| COVID Coach | ASistencia COVID-19 | BMC Combat Covid19 |
| Obvio-19 | CoronaTest Navarra | Fight Covid |
| COVID Symptom Tracker | **France** | CG Covid-19 ePass |
| PatientSphere for COVID19 | Covidom Patient | T COVID'19 |
| covid-19 Virginia Resources | **Holland** | COVA Punjab |
| Relief Central \| COVID-19 | Castor COVID-19 | COVID-19 RET |
| Disinfection Checklist | COVID-19 - Medisch DosSier | **Indonesia** |
| talli symptom tracker | COVID Radar | EndCorona |
| HEALTHLYNKED COVID-19 Tracker | **United Kingdom** | **Israel** |
| HowWeFeel | COVID-19 Sounds | המגן - האפליקציה הלאומית למלחמה בנגיף הקורונה |
| **Mexico** | StopTheSpread COVID-19 | **Kyrgyzstan** |
| Plan Jalisco Covid-19 | NHS24:Covid-19 | Stop COVID-19 KG |
| COVID-19MX | **Ireland** | **Nepal** |
| COVD-19 Cuernavaca | patientMpower for COVID-1 | Province 5 COVID-19 Tracke |
| COVID-19 Tam | Nuahealth Video Consultatios | Nepal COVID-19 Surveillance |
| **CENTRAL AMERICA-SOUTH AMERICA** | **Iceland** | **Oman** |
| **Argentina** | Rakning C-19 | Tarassud |
| CUIDAR COVID-19 ARGENTINA | **Italy** | **Pakistan** |
| **Bolivia** | ADiLife Covid-19 | COVID-19 Gov PK |
| Bolivia Segura | TreCovid19 | **Russia** |
| **Brazil** | COVID19 Regione Saredgna | Госуслуги СТОП Коронавирус |
| Telemedicina Paraná | **Lithuania** | COVID-19 онлайн тест |
| Prefeitura de Castanhal | Karantinas | Телемедицина Югры |
| Cachoeirinha ContraCoronavirus | **Poland** | **Thailand** |
| Coronavírus - SUS | mObywatel - publiczna aplikacja mobilna | DDC-Care |
| **Chile** | **Portugal** | **Vietnam** |
| cov_cl | Estamos ON - Covid19 | COVID-19 |
| **Colombia** | **Czech Republic** | FAMILY - COVID 19 |
| CoronApp - Colombia | COVID-19! - Información actual sobre infección | **AFRICA** |
| **Guatemala** | **Rumania** | **Ghana** |
| ASistencia COVID-19 GT | COVID AsSist | GH COVID-19 Tracker |
| **Jamaica** | **Turkey** | **Mali** |
| JamCOVID19 | Coronavirus Karantina Türkiye | SOS CORONAVIRUS |
| **Dominican Republic** | **OCEANIA** | **South Africa** |
| COVID-RD | **Australia** | NICD COVID-19 Case Investigation |
| **Uruguay** | Coronavirus Australia |  |
| Coronavirus UY |  |  |

**Supplementary Table 2.** Apps related to COVID-19 developed by governments and their number of downloads.

| **COUNTRY** | **APP** | **DOWNLOADS** | **COUNTRY** | **APP** | **DOWNLOADS** |
| --- | --- | --- | --- | --- | --- |
| Argentina | CUIDAR COVID-19 ARGENTINA | 500,000 | Nepal | Nepal COVID-19 Surveillance | 5,000 |
| Armenia | Covid-19 Armenia | 50,000 |  | Province 5 COVID-19 Tracker | 5,000 |
| Austria | MyAus COVID-19 | N/A^a^ | Oman | Tarassud | 50,000 |
|  | Stopp Corona | 100 | Pakistan | COVID-19 Gov PK | 500,000 |
|  | CoronaReport - COVID-19 reports for Social Science | 10 | Poland | mObywatel - publiczna aplikacja mobilna | 1,000,000 |
| Australia | Coronavirus Australia | 500 | Portugal | Estamos ON - Covid19 | N/A |
| Bahrein | BeAware Bahrain | 10 | Rumania | COVID Asist | N/A |
| Bolivia | Bolivia Segura | 50,000 | Russia | Госуслуги СТОП Коронавирус | N/A |
| Brazil | Prefeitura de Castanhal | N/A | Saudi Arabia | أمان - الأردن كوفيد-١٩ | N/A |
|  | Telemedicina Paraná | N/A |  | NICD COVID-19 Case Investigation | 100 |
|  | Coronavírus - SUS | 50,000 | Spain | Tonic: Medicina para todos | N/A |
|  | Cachoeirinha ContraCoronavirus | 1,000 |  | STOP COVID19 CAT | 500,000 |
| Canada | COVID-19 Resource for Midwives | N/A |  | ASistencia COVID-19 | 100,000 |
|  | Managing your stress & anxiety | N/A |  | COVID-19.eus | 50,000 |
|  | Canada COVID-19 | 50,000 |  | CONFINAPP | 10,000 |
|  | BC COVID-19 Support | 10,000 |  | CoronaMadrid | 10,000 |
|  | Spectrum - Clinical Decisions | 100 |  | GVA Responde | 10,000 |
| Chile | cov_cl | N/A |  | CoronaTest Navarra | 5,000 |
| Colombia | CoronApp - Colombia | 1,000.000 |  | GVA Coronavirus | 5,000 |
| Czech Republic | COVID-19! - Información actual sobre infección | 10,000 |  | AyudaCOVID | 100 |
| Dominican Republic | COVID-RD | 100 | Thailand | DDC-Care | 10,000 |
| France | Covidom Patient | 10,000 | Turkey | Coronavirus Karantina Türkiye | N/A |
| Georgia | Stop Covid - ერთად ვებრძოლოთ ინფექციას | 1,000 | United Arab Emirates | Public Access Control System | N/A |
| Germany | CoVive: your COVID-19 app | N/A |  | TraceCovid | 50,000 |
|  | COVID-19 Symptom Tracker | 5,000 |  | COVID19 UAE | 1,000 |
|  | H.A.R.D. - COVID-19 | 100 | United Kingdom | NHS24:Covid-19 | N/A |
|  | OpenWHO: Knowledge for Health Emergencies | 1 |  | COVID-19 Sounds | 1,000 |
| Ghana | GH COVID-19 Tracker | 1,000 |  | StopTheSpread COVID-19 | 100 |
| Guatemala | ASistencia COVID-19 GT | N/A | USA | Apollo COVID-19 | N/A |
| Holland | COVID-19 - Medisch DosSier | N/A |  | BeWellXcel | N/A |
|  | COVID Radar | 50.000 |  | CDC | N/A |
|  | Castor COVID-19 | 5 |  | CoronaFACTS | N/A |
| Iceland | Rakning C-19 | 50,000 |  | covid-19 Virginia Resources | N/A |
| India | BMC Combat Covid19 | N/A |  | Disinfection Checklist | N/A |
|  | COVID-19 RET | N/A |  | HowWeFeel | N/A |
|  | mCOVID-19 | N/A |  | MUSC COVID-19 Vital Link | N/A |
|  | Aarogya Setu | 50,000,000 |  | Obvio-19 | N/A |
|  | CG Covid-19 ePass | 100,000 |  | PatientSphere for COVID19 | N/A |
|  | T COVID'19 | 10,000 |  | PreMedicus | N/A |
|  | Fight Covid | 5,000 |  | Relief Central \| COVID-19 | N/A |
|  | COVA Punjab | 500 |  | talli symptom tracker | N/A |
|  | COVID-19 Tracker by Medinin | 1 |  | HEALTHLYNKED COVID-19 Tracker | N/A |
| Indonesia | EndCorona | N/A |  | patientMpower for COVID-19 USA | 500,000 |
| Ireland | Nuahealth Video Consultations | 1,000 |  | Kencor COVID-19 | 100,000 |
|  | patientMpower for COVID-1 | 1,000 |  | COVID Coach | 1,000 |
| Israel | המגן - האפליקציה הלאומית למלחמה בנגיף הקורונה | N/A |  | Sentinel Monitor (COVID-19 Management) | 500 |
| Italy | TreCovid19 | 100,000 |  | COVID Symptom Tracker | 500 |
|  | ADiLife Covid-19 | 500 |  | NIOSH PPE Tracker | 50 |
|  | COVID19 Regione Saredgna | 100 |  | corona - care | 10 |
| Jamaica | JamCOVID19 | N/A | Uruguay | Coronavirus UY | 100 |
| Korea | 자가격리자 안전보호 | N/A | Vietnam | COVID-19 | N/A |
| Kyrgyzstan | Stop COVID-19 KG | 10,000 |  | COVID-19 | 100,000 |
| Lithuania | Karantinas | 5,000 |  | FAMILY - COVID 19 | 100 |
| Mali | SOS CORONAVIRUS | N/A |  |  |  |
| Mexico | COVID-19 Tam | N/A |  |  |  |
|  | COVD-19 Cuernavaca | N/A |  |  |  |
|  | Plan Jalisco Covid-19 | 1,000 |  |  |  |
|  | COVID-19MX | 100 |  |  |  |

^a^N/A: Data on the number of downloads of the apps were obtained from the Google Play Store. These data were not available for the apps obtained from the App Store.
